# Habitat analysis of North American sand flies near veterans returning from leishmania-endemic war zones

**DOI:** 10.1186/1476-072X-7-65

**Published:** 2008-12-18

**Authors:** David Claborn, Penny Masuoka, Meredith Morrow, Lisa Keep

**Affiliations:** 1 Department of Nursing, Missouri State University, 901 South National Avenue, Missouri State University, Springfield, MO 65897, USA; 2 Department of Preventive Medicine and Biometrics, Uniformed Services University of Health Sciences, 4301 Jones Bridge Road, Bethesda, MD 20814, USA

## Abstract

**Background:**

Nearly 1300 cases of leishmaniasis have been identified in American military personnel deployed to Iraq and Afghanistan. The symptoms of this disease can range from a mild, self-limiting cutaneous infection to a deadly visceral infection and are not prevented by chemoprophylaxis or immunization. Effective treatments, however, are available. The disease-causing parasite is spread through the bite of the female sand fly. Although the disease occurs in both the Old World and the New World, the parasite species differ between the hemispheres. The large number of cases in military veterans has caused some concern that Old World, temperate-adapted parasite species could be introduced into the native sand fly populations of American military facilities where veterans of the current conflicts return following their deployments. This paper reports part of a larger study to analyze the risk of such an accidental importation. Four potential habitats on two large Army facilities in the Southeast United States were surveyed to determine relative sand fly density. The National Land Cover Map was used to provide sand fly density prediction maps by habitat.

**Results:**

Sand fly density was significantly higher in deciduous forest and even higher at the interface between forest and open grassland. The evergreen forest and agricultural fields supported very low densities. On Fort Campbell, KY, the percentage of land covered by suitable habitat was very high. A sand fly density prediction map identified large tracts of land where infected individuals would be at higher risk of exposure to sand fly bites, resulting in an increased risk of introducing the parasite to a native insect population. On Fort Bragg, NC, however, commercial farming of long leaf pine reduced the percentage of the land covered in vegetation suitable for the support of sand flies. The risk of introducing an exotic *Leishmania spp*. on Fort Bragg, therefore, is considered to be much lower than on Fort Campbell.

**Conclusion:**

A readily available land cover product can be used at the regional level to identify areas of sand fly habitat where human populations may be at higher risk of exposure. The sand fly density prediction maps can be used to direct further surveillance, insect control, or additional patient monitoring of potentially infected soldiers.

## Background

Leishmaniasis is an arthropod-borne and zoonotic disease that infects man incidentally. It is caused by *Leishmania spp*. The disease has a spectrum of manifestations, from minor, self-limiting cutaneous (skin) lesions to extreme disfigurement and death.[1] The parasite is spread to vertebrate hosts, including humans, through the bite of female sand flies. The sand fly, therefore, is the 'vector' of leishmaniasis. In the Old World, the vectors of human leishmaniasis are predominantly from the genus *Phlebotomus*. In the New World, all disease vectors are in the genus *Lutzomyia*. Parasite species also differ between the hemispheres. New World leishmaniasis is usually limited to tropical or semi-tropical environments, though a recent outbreak of cutaneous leishmaniasis in Texas demonstrates that parasites normally associated with Central America are capable of spreading into the United States.[2] Old World parasites are well adapted to transmission in temperate areas, including parts of Iraq and Afghanistan.

Between 2001 and 2006, nearly 1300 incident diagnoses of leishmaniasis were detected in veterans of the current conflicts in Iraq and Afghanistan.[3] Only four of these cases were the deadly visceral form, an infection that affects the intestines and other viscera. The rest showed cutaneous manifestations. The peak incidence rate was observed in the late summer and fall of 2003, but the disease rate has declined since. For the entire six year period, the incidence rate was 2.31 cases per 1,000 person-years. The highest risk of disease among American personnel was observed along the Iran-Iraq border where the rate exceeded 200 per 1000 deployed persons. Diagnosis can be difficult, especially in deployed settings. There are no available vaccines or prophylactic medications [4] and treatment may not eliminate all of the parasites, so infected veterans could theoretically serve as reservoirs after returning to the United States.[5]

This paper reports part of a larger study to assess the risk of introducing Old World *Leishmania *parasites into the sand fly populations of the temperate New World. The study was in three parts:

1. A survey of sand fly species on American military facilities with large numbers of soldiers deploying to Iraq or Afghanistan ;

2. A feeding study to assess the ability of a New World sand fly species to become infected with an Old World *Leishmania spp*.;

3. An analysis of sand fly abundance to identify plant communities where the risk of sand fly bites is increased.

This paper reports on the third part of the study.

Previous studies have linked adult sand fly abundance to a variety of environmental factors. Although one study noted an association between soil chemistry and the abundance of *Sergentomyia spp*.[6], most have used plant communities to identify environmental associations. In Kenya, sand fly abundance was greatest in closed canopy forests and least in thickets.[7] New World sand fly species also tended to be associated with forests. In northern Colombia, forested reserve areas displayed both greater species diversity and abundance than the degraded habitats in the same area. However, medically important species re-colonized and exploited the degraded areas, suggesting that forest degradation could lead to greater human exposure to sand fly vectors.[8] In northern Argentina, habitats were classified as primary forest, secondary forest and xeric woodland. The greatest abundance was detected in secondary forests.[9]

Possibly due to the relative lack of sand fly-borne diseases in the United States, fewer studies on sand fly ecology have been performed in this country. However, a recent outbreak of canine visceral leishmaniasis has stimulated interest in the possible role of sand flies in the transmission of disease.[10] When a focus of canine disease was investigated in upstate New York, the highest abundance of *Lu. vexator *(Coq.) was noted on steep slopes in mature mixed hardwood forests. However, on Ossabau Island off the coast of Georgia, *Lu. shannoni *Dyar was most active in established maritime live oak forests, with mixed hardwood and pine forests harboring significantly fewer sand flies.[11] In the southwest, geographic information systems were used to analyze associations with *Lu. apache*, which indicated a potential range through steppe and semi-desert vegetative provinces from Arizona to Idaho.[12]

The risk of importing exotic *Leishmania spp*. from the current war zones into the United States is dependent upon a variety of factors, including the susceptibility of North American sand flies to infection, the ability of the parasite to develop in the fly, and the availability of suitable vertebrate reservoirs. Environmental factors suitable for supporting large vector populations are also important variables. This part of the study attempts to identify and locate those variables in areas where returning, and possibly infected, veterans could come into contact with sand fly vectors.

## Methods

Three military facilities with large populations of deploying soldiers were initially selected for the study. The facilities (Fort Hood, TX; Fort Bragg, NC and Fort Campbell, KY) each represented a different dominant plant community considered suitable for supporting sand flies. Unusual weather patterns in the summer of 2007 destroyed most of the trapping sites on Fort Hood, so only Fort Campbell and Fort Bragg are reported here. Twenty sites from a variety of vegetation communities were selected on each facility. Weekly sampling of sand fly activity on each site was conducted using a CO_2_-baited CDC light trap with a protective cover. The contents of each trap were collected before 0900 each morning, packed in padded boxes and mailed overnight to the Uniformed Services University of Health Sciences in Bethesda, MD. The sample was frozen until it could be microscopically examined and sorted. Sand flies were placed in a lacto-phenol clearing solution for at least 30 days, mounted on slides and identified according to published keys.[13]

### Study sites

Fort Bragg is in Cumberland and Hoke Counties of North Carolina and covers 65,165 ha. The primary forest type is the long leaf pine (*Pinus palustris*). Hardwood undergrowth is rigorously suppressed with an active controlled burn program as part of an endangered species program, so much of the forested area is monocultural. The forest is highly managed for commercial logging, with extensive efforts in timber, pine cone and pine straw harvesting.

Fort Campbell straddles the borders of Kentucky and Tennessee in the rolling Pennyroyal Plain. It consists of approximately 42,510 ha that experienced nearly complete deforestation by the mid-19^th ^century. Regrowth forest includes the following species: oak (*Quercus spp*.), hickory (*Carya spp*.), sassafras (*Sassafras albidum*), persimmon (*Diospyros spp*.) and eastern red cedar (*Juniperus virginiana*) When pine trees are present, they are typically loblolly pine (*Pinus taeda*) and are in forest management programs.

Trapping sites were located precisely by GPS and were selected to represent one of four general habitats: deciduous forest, evergreen forest, agricultural land or forest/grassland interface. Because the traps were hung on available trees, the grassland sites were considered to be the transitional area between the forest and grassland (an ecotone). Safety and access issues prevented the selection of an equal number of sites for each habitat surveyed.

### Statistical analysis

The trapping rate for each site was determined by dividing the total number of sand flies by the number of trap-nights for the entire study period (Summer, 2007). The sites were ranked according to rate, then compared using a Kruskal-Wallis test [14] with vegetative cover types as treatment categories.

### GIS Analysis

Sand fly density prediction maps were created using data from the National Land Cover Database 2001 (NLCD 2001). The NLCD is a 30 meter pixel resolution land cover classification of the United States developed from Landsat satellite imagery.[15,16] NLCD 2001 follows the NLCD 1992 but uses improved algorithms for processing. The data set was created by the Multi-Resolution Land Characteristics (MRLC) Consortium-a group of federal government agencies that purchase Landsat images and create land cover products for use by the government and the general public. The data set is available for download from . For simplification of the maps, land cover classes on the NLCD map were combined into functional habitat categories as shown in Table 1.

**Table 1:** Functional habitat categories

NLCD Classes	Combined Classes
Water	Water
Developed, open space	Urban
Developed, low intensity	Urban
Developed, medium intensity	Urban
Developed, high intensity	Urban
Barren land, rock/sand/clay	Bare
Deciduous Forest	Deciduous
Woody wetlands	Deciduous
Evergreen Forest	Evergreen
Mixed Forest	Mixed Forest
Shrub/scrub	Shrub/Scrub
Grassland/herbaceous	Grass/Crop
Pasture/hay	Grass/Crop
Cultivated crops	Grass/Crop

The woody wetlands category corresponded to deciduous trees growing along stream valleys at Fort Bragg and were thus assigned to the deciduous class.

Sand fly density prediction maps for the two bases were created in ArcGIS using land cover derived from the NLCD. Several steps were required to produce these maps. In the first step, the land cover maps were converted from a raster (TIFF format) to a vector format (shape file). Next, the land cover classes associated with high densities of sand flies were extracted and saved as separate deciduous and grass/crop shape files. To map the ecotones, the two shape files were used to perform a geographical intersection in ArcGIS with the output consisting of the common line between the two sets of polygons. Fifty meter buffer zones were created around the resulting intersection lines to represent the areas of highest sand fly density at the ecotones. A fifty meter buffer was selected for the buffer zone size based on the estimated flight range of the sand fly. The final prediction map, uses the buffer zone as the highest density and the deciduous land cover as medium density for sand flies. The urban land cover classes are also displayed on the map in order to provide a spatial reference to the map user.

## Results and discussion

Sand fly abundance on Fort Bragg was extremely low, yielding only 31 flies for the entire summer of 2007. The low number of sand flies prevented a comparison between habitats, but it is not surprising given the degree to which Fort Bragg is covered by a highly managed long leaf pine plantation. This habitat has previously been reported as supporting very low numbers of sand flies [11], so the risk of human exposure to sand fly bites on Fort Bragg is relatively low. Fort Campbell, however, was very different.

Table 2 describes the relative abundance of sand flies in four different habitats on Fort Campbell, KY as measured by carbon dioxide-baited CDC light traps. The trap rates for *Lu. shannoni *are the only ones presented because this species is the primary human feeding fly. The relatively low densities in pine forest and agricultural lands are consistent with previous studies which demonstrated higher abundance in deciduous forests. These results, however, indicate that the ecotone transition from deciduous forest to open grassland supports the greatest abundance of *Lu. shannoni*. The concentration of vector populations in ecotones has been noted with other vectors; however, this is the first such report with North American sand fly species.[17,18]

**Table 2:** Mean number of Lutzomyia shannoni trapped in twenty sites from four different habitats on Fort Campbell, KY (Summer, 2007)

Habitat	Mean trap rate	# of sites	Mean rank	# of trap-nights
Evergreen trees	0.30	2	3.5a^1^	23
Agricultural fields	0.46	2	5.0ab	24
Deciduous trees	1.53	12	10.7b	224
Forest/meadow interface	3.98	4	16.1c	48

1 Mean for each site compared with Kruskal-Wallis rank test (p = 0.05). Values followed by the same letter are not significantly different.

**Figure 1:**
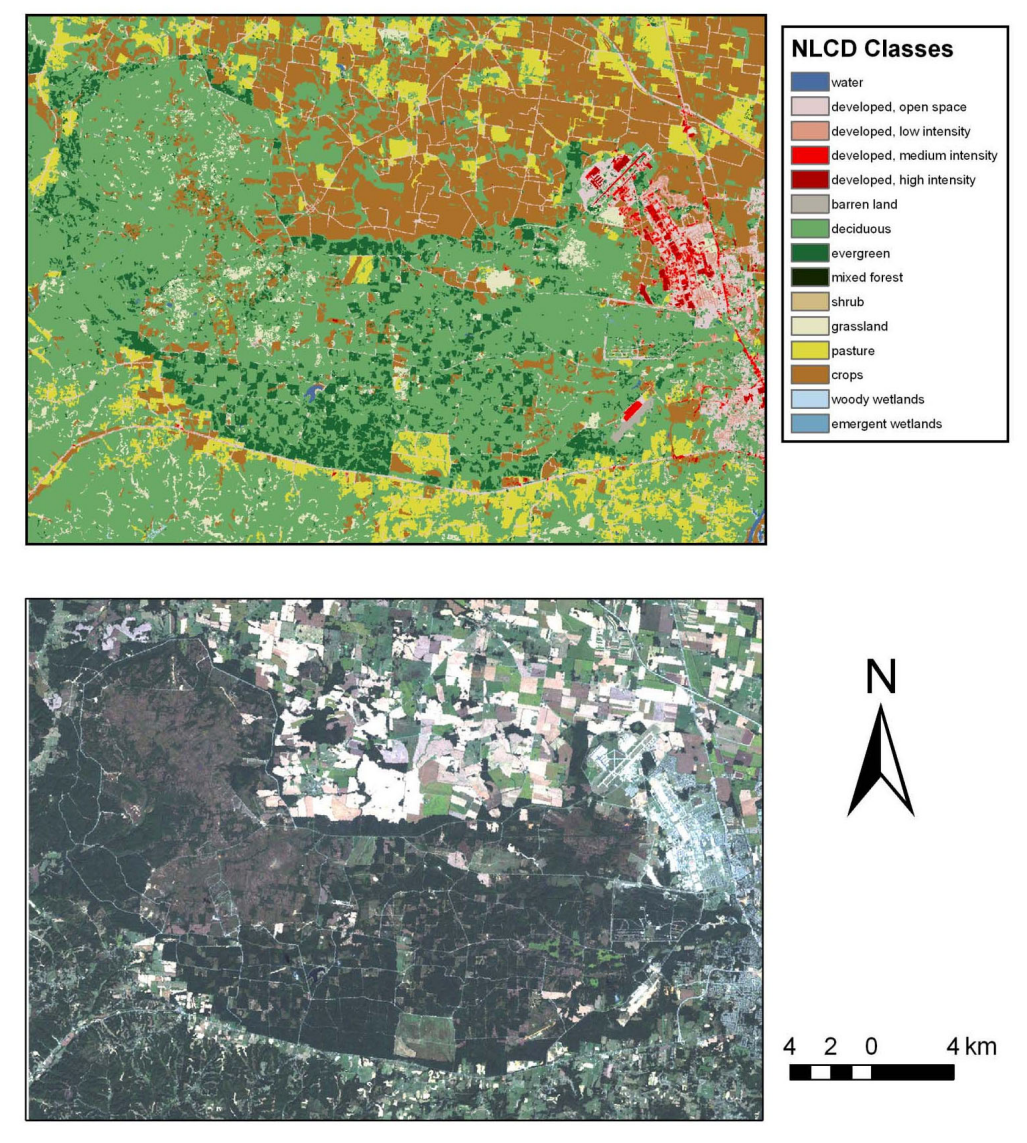
**Two images of Fort Campbell, KY indicating the vegetative cover on the facility and surrounding vicinity**.

**Figure 2:**
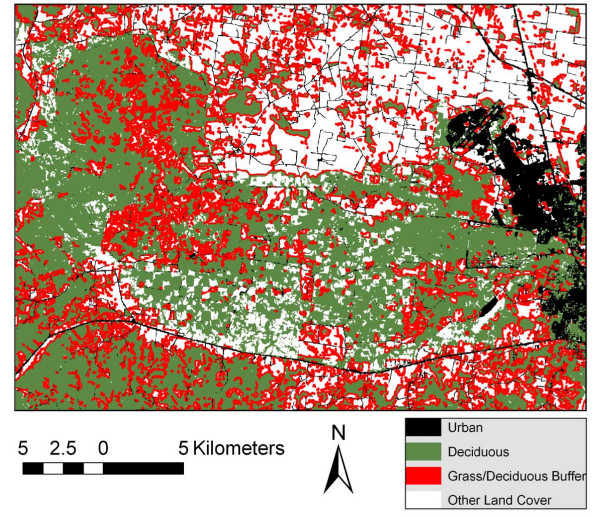
**A reclassification of the NLCD image showing functional classifications of sand fly habitats on Fort Campbell**.

Figure 1 displays the typical land cover for Fort Campbell as depicted by real color and by the NLCD image. The lower image is a Landsat product acquired on September 29, 2001, depicting Fort Campbell as the darker, irregularly shaped central image surrounded by the lighter agricultural fields. The upper figure is the NLCD land cover classification of a Landsat image. The number of ground cover categories was too complex for analysis, so a simplified reclassification of the NLCD image is displayed in Figure 2. It represents broad categories of potential sand fly habitats: urban, deciduous forest, forest/grassland ecotone and other. The image provides a sand fly density map based on our trapping results and serves as a 'risk map' for encountering *Lu. shannoni *on Fort Campbell. In particular, the green areas (deciduous forests) and red areas (50-m buffer along the forest/grassland ecotone) depict habitats that support greater densities of sand flies. This classification has public health utility because it identifies areas where possibly infected veterans returning from Iraq or Afghanistan are more likely to encounter native American sand flies, in particular *Lu. shannoni*. Where the red areas are contiguous to black (urban) areas, the risk of human exposure with sand fly bites is considered to be increased. Such an encounter could result in the accidental introduction of an Old World parasite into the New World vector population, where it could subsequently be introduced to a native mammal reservoir population as well.

**Figure 3:**
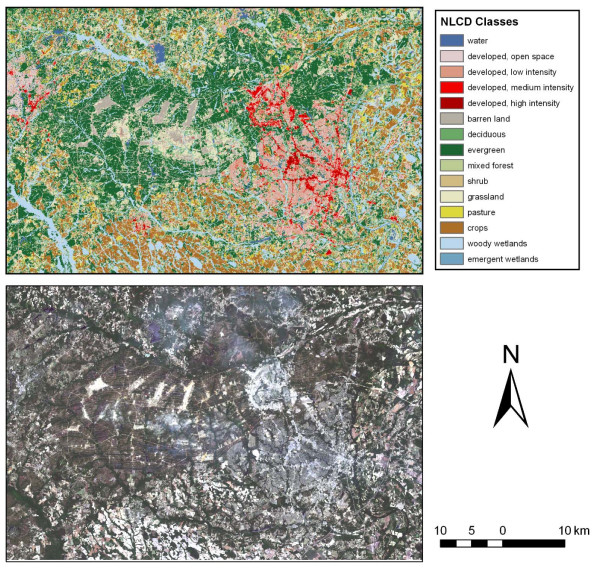
**Two images of Fort Bragg, NC indicating dominant land cover types**.

**Figure 4:**
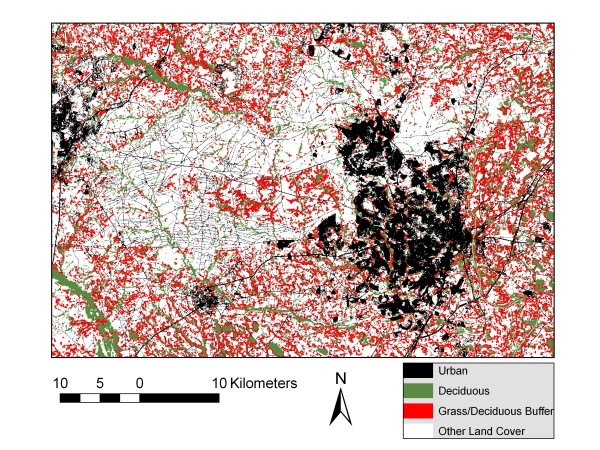
**A reclassification of the NLCD image of land cover on Fort Bragg, NC**.

**Figure 5:**
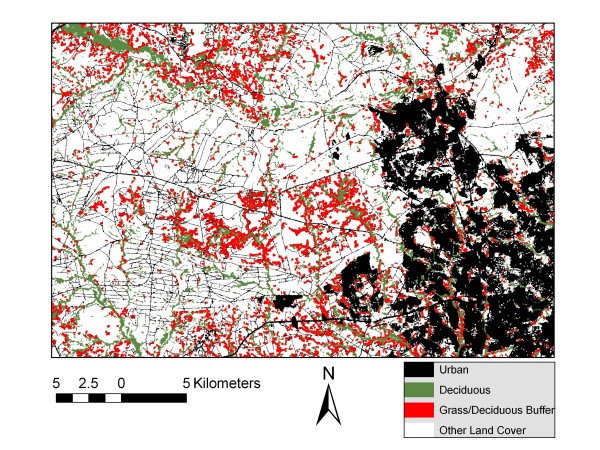
**An enlargement of Figure **4** showing areas where sand fly habitat abuts urbanized vicinities**.

Figure 3 displays typical land cover for Fort Bragg, NC, and it reveals a very different environment. The lower image is a real color Landsat image acquired on 24 May, 2002; the upper image is the NLCD land cover product of the same area. Fort Bragg is seen as the largely dark green central image with red urban areas toward the right side. The NLCD image clearly depicts the pine forest on Fort Bragg, a habitat which supports relatively low populations of sand flies in North America. Table 3 quantifies the differences in ground cover between the two facilities by comparison of the percentage of pixels in the NLCD representing the different ground covers. The percentage of land covered by evergreen forest on Fort Bragg was four times that on Fort Campbell, but only a third as much deciduous forest occurred on the former. This difference in land cover probably played a significant role in the much lower number of sand flies on Fort Bragg. The residual deciduous forests on Fort Bragg were associated with creek bottoms and other drainage sites. This type of forest was easily detected in Figure 4, a reclassification of the NLCD image using the same four broad vegetation categories used in Figure 2. Fort Bragg is seen as the mostly white irregular image in the center with urban areas, depicted in black, on the right. The smaller area of high risk deciduous and forest/grassland ecotone is due to the much greater area dedicated to pine plantations on Fort Bragg as compared to Fort Campbell. The 'other' category in white includes the pine forests. The large amount of white space in Figure 3 depicts these evergreen forests where sand fly populations are low and risk of exposure and introduction of exotic parasites are also low. Figure 5 is an enlargement which identifies areas where high density sand fly areas (red and green) are located near urban areas (black). These urban areas include office space, housing, shopping and training centers. Where these areas of sand fly habitat and high human activity are contiguous, the risk of exposure is obviously increased.

**Table 3:** Amount of each NLCD 2001 land cover class on the Fort Campbell, KY and Fort Bragg, NC classifications

FORT CAMPBELL, KY		
		
Class	Number of Pixels	Percent of Total Land Cover
		
Water	1595	0.1424%
Urban	90189	8.0513%
Bare	1476	0.1318%
Deciduous	569108	50.8050%
Evergreen	76060	6.7900%
Shrub	798	0.0712%
Grass/Crop	380956	34.0084%
		
FORT BRAGG, NC		
		
Class	Numberof Pixels	Percent of Total Land Cover
Water	32518	0.9813%
Urban	533786	16.1082%
Bare	54129	1.6335%
Deciduous	619454	18.6934%
Evergreen	980612	29.5922%
Mixed	66431	2.0047%
Shrub	66173	1.9969%
Grass/Crop	960649	28.9898%

These sand fly density maps provide a planning document for preventing the introduction of an exotic parasite and can be used to direct the following actions:

1. Additional sand fly and vertebrate reservoir surveillance in high risk areas where infected veterans live or take part in field exercises;

2. Vector control efforts to reduce human exposure to sand fly bites;

3. Increased monitoring of returning veterans living or working in high sand fly risk areas.

## Conclusion

Sand fly populations on Fort Campbell were highly associated with deciduous forests and the transitional ecotone between forest and grassland. This information can be used to construct sand fly density prediction maps for affected areas. These density maps can then be utilized to assess the risk of introducing an exotic parasite with returning veterans. They can also be used to direct insect surveillance, insect control and infestion status monitoring of veterans living near suitable sand fly habitat.

## Competing interests

The authors declare that they have no competing interests. Toward full disclosure, two of the authors (DC and LK) are commissioned officers in the military of the United States of America. Funding for this research was provided by an internal grant from the Uniformed Services University of Health Sciences in Bethesda, MD.

## Authors' contributions

DC designed the experiment, conducted the field research, identified all specimens, performed the statistical analysis and served as the co-primary investigator and primary author. PM and MM performed all GIS analysis of the data; and obtained, reclassified and interpreted all images and results. LK served as the co-primary investigator, obtained all funding and contributed as an author.
